# Biowaste as a Potential Source of Bioactive Compounds—A Case Study of Raspberry Fruit Pomace

**DOI:** 10.3390/foods10040706

**Published:** 2021-03-26

**Authors:** Slađana Krivokapić, Milorad Vlaović, Biljana Damjanović Vratnica, Andrej Perović, Svetlana Perović

**Affiliations:** 1 Department of Biology, Faculty of Natural Science and Mathematics, University of Montenegro, Podgorica 81000, Montenegro; vlaovicmilo@gmail.com (M.V.); andrejp@ucg.ac.me (A.P.); svetlanap@ucg.ac.me (S.P.); 2 Faculty of Metallurgy and Technology, University of Montenegro, Podgorica 81000, Montenegro; biljanad@ucg.ac.me

**Keywords:** antioxidants, polyphenols, HPLC, byproduct

## Abstract

Raspberry fruit pomace, a byproduct of juice production, was studied as a potential source of antioxidant compounds. Target high-performance liquid chromatography analysis of important polyphenolic compounds (gallic, *p*-coumaric, caffeic, quercitrin, chlorogenic, and ellagic acid) was performed together with analysis of total phenolic content (TPC), total flavonoid content (TFC), total anthocyanins content (TAC), and antioxidant capacity (via ferric reducing antioxidant power (FRAP) and 1,1-diphenyl-2-picrylhydrazyl (DPPH) assays). The differences in polyphenolic content of *Rubus idaeus* L. pomace were evaluated following ultrasound-assisted extraction and conventional maceration with different organic solvents. Additionally, the yield of free phenolics was measured in hydrolyzed pomace extracts. The results obtained show that the ultrasound method maximizes the quantity of antioxidant compounds in terms of TPC (27.79 mg/L gallic acid equivalent (GAE)), TFC (8.02 mg/g quercetin equivalent (QE)), TAC (7.13 mg/L cyanidine-3-glucoside equivalent (C3G Eq)), caffeic (19.17 µg/g), chlorogenic (3.56 µg/g), ellagic (105.52 µg/g), and gallic acids (8.75 µg/g), as well as FRAP (1002.72 µmol/L) and DPPH assays (969.71 µmol/mL vitamin C equivalent (vit C Eq); 567.00 µmol/100 g Trolox equivalent (TE)). On the other hand, conventional maceration maximizes the yield of quercetin and *p*-coumaric acid. In terms of biowaste valorization, raspberry fruit pomace has a promising industrial potential and may prove to be useful in the development of antioxidant dietary supplements.

## 1. Introduction

The impairment of metabolic and other life-sustaining molecular processes leads to significant oxidative stress and the production of reactive oxygen species. Additional oxidative stress is introduced by environmental stressors and even common air pollution [1,2,3]. Prolonged oxidative stress in humans may result in the onset and exacerbation of any of the plethora of conditions associated with oxidative stress, such as premature aging, cardiovascular diseases, chronic obstructive pulmonary disease, neurodegenerative diseases such as Alzheimer’s or Parkinson’s disease, various forms of cancer, and sarcopenia. Oxidation is a process heavily involved in the spoilage and perishability of food and drugs, particularly lipid peroxidation, which also undesirably affects the flavor principles [4,5,6].

Adequate fruit and vegetable intake (more than 10 servings per day) is epidemiologically justified as a strategy to counter the effects of oxidative stress and inflammation that are associated with heart diseases and diabetes—both of which have a high mortality rate worldwide. Fruits and vegetables accomplish this due to their secondary metabolites, which exhibit antioxidant, anti-inflammatory (especially the triterpenoids and phenols), anticarcinogenic, and other biological effects [7,8,9]. Most fruit (and vegetable) loss and wastage occurs during processing. The fruit pomace accounts for a significant part of fruit and vegetable waste and losses (FVWL), reaching millions of metric tons (MMT) of waste per year. Up to 5.5 MMT of waste is created during juice production [10]. Secondary metabolites, such as fibers, carotenoids, anthocyanins, and phenols, are wasted, along with the fruit pomace, during production [11]. Among others, these compounds have antioxidant, antimicrobial, and UV-protective properties, and might serve as natural preservatives, additives, emulsifiers, thickeners and bulking agents, dyes/colorants, etc. [12]. This is particularly important when considering the increasing consumer trend of rejecting their synthetic counterparts. FVWL also poses an unnecessarily exaggerated challenge to waste management in the food and nutraceuticals industry. Bioactive compounds, readily found in fruit biowaste, offer low-cost, integrated, and environmentally friendly alternatives to their chemically synthesized counterparts, and are an environmentally conscious choice with promise as a tool in the achievement of a sustainable circular economy [10,11,12,13,14].

Raspberry fruit is naturally rich in antioxidant compounds, anthocyanins, ellagitannins, and fiber [15]. Around 80% of its content is water, and the predominant macronutrients are carbohydrates. However, the chemical properties strongly depend on the edaphoclimatic conditions of the area where given raspberries were grown [16]. Although Spain is a leader in raspberry fruit export, a great number of raspberries sold worldwide are sourced from South-Eastern Europe and represent a fast-growing segment of the local horticulture and food industries [17,18]. However, increases in fruit production lead to an increase in fruit waste and losses [11].

During juice production, most antioxidants are wasted with the raspberry fruit pomace (RFP). It is reported that RFP contains 77.5% of the total dietary fiber present in the fresh fruit. A large amount of phenols remains in the RFP as well. The seeds present a good source of tocopherols and λ-linoleic acid. With the discarded RFP, a significant biological potential is lost as well in terms of the antioxidant, antiproliferative, and antihyperglycemic effects of RFP [11,19,20]. In general, RFP is not extensively investigated in Europe, compared with the whole raspberry fruit and juice, in terms of chemical content and possible reuse [21,22]. Only a few studies conducted in some parts of South-Eastern Europe (such as Serbia) are available [20,23,24,25]. On the other hand, the phytochemical profile and biological activity of RFP obtained from plants harvested in Spain in the Mediterranean region are well documented. 

The most applied extraction method for the investigation of berry fruits and byproduct biopotential is conventional maceration due to the ability to “spare” heat-sensitive compounds, as well as the low cost, wide availability, and efficiency [26]. However, novel and unconventional methods that require less energy and solvent consumption are attracting growing attention, such as enzyme- and microwave-assisted extraction, pulsed electric field extraction, and ultrasound-assisted extraction (UAE). Nowadays, UAE is identified as a rapid, more efficient technique for the extraction of phenols and anthocyanins from plant material, by causing cavitation via ultrasound waves in plant cell walls. In all of these methods, extraction seems to be further amplified if solvents are mildly acidulated, usually at a 1% amount, with HCl, CH_3_COOH, or other acids [11,27]. 

Therefore, in order to expand the existing knowledge on the biological potential of raspberry fruit pomace, the aim of this research was to investigate the total phenolic, flavonoid, and anthocyanin content, as well as the antioxidant activity, of *Rubus idaeus* L. pomace harvested in the Mediterranean region, namely Montenegro. Ultrasound-assisted extraction and conventional maceration were applied as extraction techniques and their efficiency was compared. Moreover, the polyphenolic profile of obtained extracts was evaluated using high-performance liquid chromatography analysis. 

## 2. Materials and Methods

### 2.1. Plant Material and Sample Preparation

The raspberry (*Rubus idaeus* L., *Rosaceae*) fruits, harvested at Ravna Rijeka (43.1219° N, 19.6500° E), a village in the Municipality of Bijelo Polje, in Montenegro, were collected during the summer of 2019. The fruits were used for juice extraction, using a destoning machine, and upon extraction the remaining pomace was packed in clean plastic containers and kept frozen at −4 °C until further use. 

Raspberry pomace (2 g) was extracted by two different methods: ultrasound-assisted extraction (UAE) and conventional maceration (CM). Following homogenization of the pomace with 10 mL of an aqueous solution of mildly acidulated methanol, containing formic acid (1%) and methanol (80%), UAE was performed using an ultrasonic cleaner (Vims Elektrik) for 120 min at 50 °C and 50 kHz. The CM extraction was performed by adding 10 mL of 80% methanol (without acidification) to the pomace sample for 120 min at 20 °C. Both the UAE and the CM extracts were then centrifuged at 4000 rpm for 20 min. Supernatants were carefully decanted and filtered through a Whatman grade 597 filter paper into clean glass vials. Finalized extracts were then kept in a fridge, at 4 °C, until further analysis. 

### 2.2. Determination of the Total Phenolic, Flavonoid, and Anthocyanin Content

The total phenolic content (TPC) of the pomace extracts was evaluated by the colorimetric Folin–Ciocalteu method as described by Singleton and Rossi [28], with minimal modifications. The results were expressed as the mass of gallic acid equivalent (GAE) per volume of extract (mg/L GAE). 

The total flavonoid content (TFC) of the pomace extracts was determined by the aluminum chloride colorimetric method according to Chandra et al. [29], using quercetin as a standard. The results were expressed in mg quercetin equivalent per gram of pomace (mg/g QE). 

The total anthocyanin content (TAC) of the pomace extracts was determined by the pH differential method according to Giusti and Wrolstad [30]. Concentrations were expressed as cyanidine-3-glucoside (C3G) equivalent in mg/L. 

### 2.3. Antioxidant Assays

The ferric reducing antioxidant power assay (FRAP) was conducted according to the original protocol developed by Benzie and Strain [31]. The antioxidant potential was expressed in µmol/L FRAP. Two 1,1-diphenyl-2-picrylhydrazyl (DPPH) free radical scavenging methods were used. Based on the method of Brand-Williams et al. [32], the absorbance (at 517 nm) obtained from *L*-ascorbic acid standard solutions reacted with the DPPH was used to produce a standard curve. The obtained results were expressed as µmol/mL vitamin C (vit C) equivalent. IC50 (half maximal inhibitory concentration) was calculated from the antioxidant activity curve by linear regression. The DPPH assay method by Blois [33] was also used, and the results were expressed as μmol/100 g Trolox equivalents (TE).

### 2.4. Determination of Polyphenolic Content

The chemical characterization of the examined extract and the quantification of the selected compounds were performed before and after hydrolyses using an Agilent Technologies 1100 liquid chromatographer equipped with a diode-array detector (Agilent Technologies, Santa Clara, CA, USA). Gallic, *p*-coumaric, caffeic, quercitrin, and chlorogenic acid were analyzed using a validated high-performance liquid chromatography (HPLC) method by Salaj et al. [34]. Ten microliters of pomace extract were injected and separation was performed using a reversed-phase Nucleosil C18 column (250 mm × 4.6 mm, 5 μm particle size; Agilent Technologies) held at 30 °C. The mobile phase consisted of solvent A (0.1% (*v*/*v*) aqueous HCOOH with 10 mmol CH_3_COONH_4_) and solvent B (pure methanol). The mobile phase was delivered in the gradient mode (0 min 10% B, 10 min 25% B, 20 min 45% B, 35 min 70% B, 40 min 100% B, 46 min 10% B) using a variable flow rate (0–10 min, 1 mL·min^−1^; 10–20 min, 0.8 mL·min^−1^; 20–30 min, 0.7 mL·min^−1^; 30–46 min, 1 mL·min^−1^). The total run time was 48 min. 

Ellagic acid was quantified using a Zobax SBC18 column (250 × 4.6 mm i.d.; 5 μm) according to Zhou et al. [35]. Ten microliters of pomace extract were injected and the mobile phase consisted of methanol, ethyl acetate, and potassium dihydrogen phosphate-phosphoric acid (both at 0.05 M) in the ratio 34:2:64 (by volume). Ellagic acid was detected at 254 nm using a constant flowrate of 1.0 mL/min at 30 °C. For more analytical details, see Salaj et al. [34] and Zhou et al. [35].

### 2.5. Statistical Analysis

All measurements were conducted in triplicate and the values were expressed as mean ± standard deviation (SD). The statistical analysis of obtained experimental data was performed using Statistica v. 12.5 software (StatSoft, Tulsa, OK, USA). The obtained dataset was analyzed by a multivariate statistical method of hierarchical cluster analysis (HCA), which was performed on squared Mahalanobis distances.

## 3. Results and Discussion

### 3.1. The Total Phenolic, Flavonoid, and Anthocyanin Content of RFP 

In this work, the content of antioxidants was determined, via the TPC, TFC, and TAC in vitro assays (Table 1), and compared with the available literature data (Table 2). The sample that underwent UAE demonstrated higher phenolic, flavonoid, and anthocyanins content than the CM sample (Table 1).

The obtained results are in good agreement with the previously published data from studies that investigated RFP and lyophilized fruit extracts (which ranged from 2.34 to 43.43 mg/g GAE for TPC and 5.19 to 16.35 mg/g QE for TFC). However, the TAC values in the analyzed RFP using both extraction methods showed better results than those of raspberry pomace extracted from lyophilized fresh (29.69–81.13 mg/100 g C3G EQ), as well as fresh-frozen raspberry fruit (144.55 mg/100 g C3G) extracted by maceration with 80% methanol [36,38]. The TPC values determined for RFP extracts are similar to those reported by Brodowska [19] (591.69 mg/100 g fresh pomace) and are in good agreement with blackberry (*Rubus fruticosus* L.) pomace TPC results [37,39]. The samples analyzed had higher TAC than enzymatically treated raspberry juice did (0.09–0.12 g/L) [37], but were within the range of microfiltered blackberry juice (5.6 mg/g) [41]. 

It is worth noting that since microfiltering is expensive and logistically demanding, UAE and CM may be methods of choice for extraction if the resulting concentrations of the targeted compounds in the sample/extract are approximate to those obtained by microfiltering. The determined TPC and TAC values in this work are slightly greater than those obtained from fresh fruits by pressing (2.62–3.81 mg/100 g GAE and 0.08–0.18% C3G, respectively) [42]. The TPC and TAC levels in this work were found to be within the range of several cultivars (from the genera: *Rubus* (raspberry and blackberry), *Ribes*, and *Vaccinium*) whole fruit values [38,43,44]. Considering the obtained results, RFP can yield similar phenolic and anthocyanin contents as juice, or even the whole fruit in lyophilized form, particularly if these compounds are extracted ultrasonically with an aqueous solution of mildly acidulated methanol. 

The phenolic content can vary even among specimens of the same plant species, and the results of TPC are influenced by environmental factors such as climate, coastal proximity, soil, etc. Some plant species found in colder climates, at higher altitudes, and growing in a more arid environment tend to produce more phenols than the same species growing under different conditions [45,46]. The studied raspberry (*Rubus idaeus* L.) tends to fare better in colder climates and higher altitude and this probably accounts for the relatively high TPC found in the pomace samples analyzed. The collected *Rubus idaeus* L. was harvested in the northern part of Montenegro (Ravna Rijeka) at 655 m altitude, growing on dystric cambisol (which can get excessively drained) under the influence of continental and highland climate. Montenegro belongs to the Mediterranean region and is considered an ecological country. The region has a very specific, opulent range of plant species (around 2000), roughly half of which are endemic, providing a sort of “bridge” between the species (and their areal distribution) of the tropical and temperate flora [47]. 

In this work, TPC was three times higher after UAE than after CM. According to the literature findings: ultrasound techniques are more suitable for the extraction of polyphenols from fruit waste (such as peels and pomace), they require lower temperatures, quantities of solvents, and favor solubilization of targeted compounds [48,49]. Based on the results obtained, UAE combined with acidulated 80% methanol contributed to better extraction of TPC, TFC, and TAC, compared with extraction using CM and simple 80% methanol aqueous solution. This was expected, considering the findings in the literature about the effect of solvent acidification on the extraction of phenols from fruit waste [27,50].

### 3.2. Antioxidant Activity of RFP

Both the DPPH and the FRAP assay used in this work to determine the antioxidant activity of RFP extracts indicated higher antioxidant activity in the UAE sample (Table 3). The obtained data were compared with the available literature data (Table 4). 

The sample extracted by CM displayed lower antioxidant activity in both assays—772.73 compared with 1002.72 μmol/L (for the UAE sample) in the FRAP assay. The DPPH IC50 for the UAE sample is nearly two times lower than that of the CM sample, and the DPPH activities in TE of the given samples were: 567.00 TE μmol/100 g and 361.28 TE μmol/100 g for UAE and CM, respectively. 

The obtained UAE extract showed higher DPPH and FRAP values and significantly higher content of redox-active compounds (TPC, TFC, and TAC). This fact is not surprising considering the reports in the literature about the positive correlation between the content of redox-active compounds—particularly the phenols and anthocyanins of berry extracts—and DPPH and FRAP in vitro assays [45]. The DPPH IC50 values of samples in this work were higher than those reported in enzymatically treated anthocyanin fractions of the raspberry pomace [54]. In general, TPC, TFC, and particularly TAC, as well as their respective antioxidant activities in terms of DPPH and FRAP assays, appear to be higher in the raspberry fruit extracts obtained with the help of ultrasound (UAE) in contrast to simple conventional maceration (CM) [51,52,55].

In addition, the FRAP values (μmol/L) obtained in this work indicate that the sonication process is desirable in achieving higher antioxidant activity. The determined FRAP values are in good agreement with those for various other fruits and vegetables [53]. Methanol as a solvent of choice seems to yield the highest antioxidant activities, compared with other alcohols, in the DPPH in vitro assays [56]. Much like in the case of extraction of phenols, flavonoids, and anthocyanins, if solvent was acidulated with a small volume of acid (1%), as was done with the UAE sample in this work, it tended to result in higher values of the antioxidant activity. Acidulation of the solvent, therefore, in this work appeared to enhance the antioxidant activity of the sample, much like it is reported to in the literature, and contributed to the variation in the samples’ biological activity [27]. 

### 3.3. The Polyphenolic Content of RFP

Due to the presence of a large number of flavonoid glycosides, quantification of individual flavonoid glycosides is difficult. Additionally, the availability and cost of standards for these glycoside forms of flavonoids make it difficult to quantify these in complex plant extracts. These points make a strong base for the need of hydrolysis to make the aglycon part free from the glycoside part [57]. 

Target HPLC analysis of important polyphenolic compounds: gallic, *p*-coumaric, caffeic, chlorogenic, ellagic acid, and quercitrin, was performed on the RFP samples. It demonstrated that UAE samples, as well as CM samples, yielded lesser content of these compounds (in µg/g extract) if the sample underwent hydrolysis, with the exception of quercetin. Quercetin content was independent of hydrolysis in the UAE sample (Table 5).

The ellagic acid content of the RFP samples was found to be significantly higher than the content of other polyphenolic compounds, which is in accordance with commonly reported results in the literature which demonstrated that ellagic acid constituted up to 50% of total phenols in raspberry fruit [58,59]. The RFP without seeds is reported to have around 12% of the total ellagic acid content of the fruit (~0.18 mg/g or 180 µg/g), whereas raspberry juice contains only a negligible amount [60]. Considering the well-reported, diverse biological effects (antioxidant, anti-inflammatory, antiglycemic, proestrogenic, antiestrogenic, prebiotic, antimutagenic, and anticarcinogenic) of ellagic acid, RFP is a rich source of this valuable compound [61]. The content of ellagic acid in the UAE pomace sample in this work is very similar to that reported by Daniel et al. [60]. 

Apart from the ultrasound, the increased temperature, and acidification of the solvent, all other extraction parameters, such as concentration of solvent and solid/liquid ratio, were the same. Based on the obtained results and in terms of antioxidative activity and polyphenolic content, the increase in temperature, together with the acidity of the solvent and sonification, significantly improved the efficiency of bioactive compound extraction from the RFP.

### 3.4. A Multivariate Statistical Analysis

**Figure 1:**
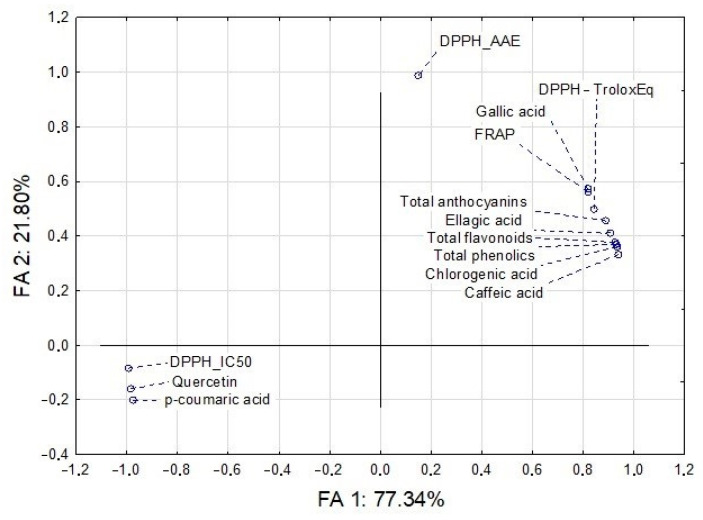
Factor analysis of the chemical profile and antioxidant potential of the raspberry pomace extracts.

The application of multivariate analysis (factor analysis) on the dataset describing the chemical profile and antioxidant potential of the raspberry pomace extracts showed that, after the extraction of principal components and varimax normalized rotation, the first two factor axes (Figure 1) describe more than 99% of the sample’s variability. In terms of factor axis 1 (FA 1), the variability of samples is significantly described by the quantified amounts of *p*-coumaric, quercetin, and by the results of antioxidant potential obtained in DPPH assay (DPPH-IC50). On the other side, the shape of the variability is mostly determined by factor axis 2 (FA2), and significantly correlates with the results obtained in the DPPH assay, expressed as ascorbic acid equivalents (DPPH_AAE). All other variables, such as caffeic, gallic, ellagic and chlorogenic acids, the amounts of total phenolics, total flavonoids, and total anthocyanins, as well as the results of antioxidant potential obtained in FRAP and DPPH assays (DPPH-Trolox Eq), had a strong mutual correlation (parameters grouped together) and are described by both factors (FA1 and FA2). 

**Figure 2:**
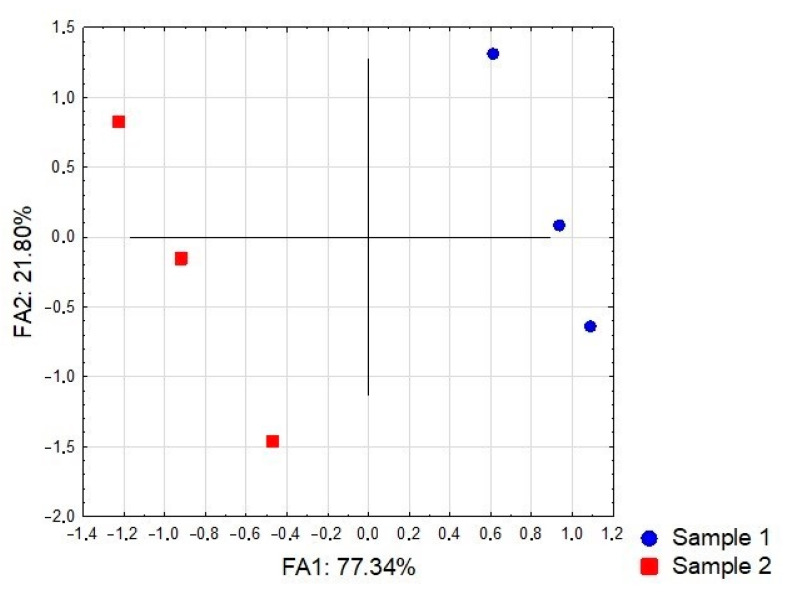
The position of the examined samples in the space defined by the first two factor axes.

Figure 2 reveals grouping of the UAE sample (Sample 1 in Figure 2) in the positive part of FA 1 as a result of the higher content of total phenolics, flavonoids, and anthocyanins, gallic, caffeic, chlorogenic and ellagic acids, as well as significantly higher antioxidant potential, exhibited in all of the assays performed. On the other hand, localization of the CM sample (Sample 2 in Figure 2) in the negative part of FA 1 is a consequence of higher quercetin and *p*-coumaric acid levels, as well as weaker antioxidant potential, when compared with the UAE sample.

Phenolic compounds, for example *p*-coumaric acid, are reported to be more highly correlated with antioxidant capacity than anthocyanins are [52]. Interestingly, in this work, based on the factorial analysis, this compound is grouped with the DPPH assay IC50 values. 

## 4. Conclusions

Based on the obtained results, ultrasound-assisted extraction (UAE) of the raspberry pomace maximized the yield in terms of total phenolics (27.79 mg/L GAE), flavonoids (8.02 mg/g QE), and anthocyanins (7.13 mg/L C3G Eq), particularly caffeic (19.17 µg/g), chlorogenic (3.56 µg/g), ellagic (105.52 µg/g), and gallic acids (8.75 µg/g). Moreover, UAE ensured higher antioxidant potential of the obtained extract, in comparison with conventional maceration (CM), in terms of FRAP (1002.72 µmol/L) and DPPH assays (969.71 µmol/mL vit C Eq; 567.00 µmol/100 g Trolox Eq). On the other hand, maceration maximizes the yield of quercetin and *p*-coumaric acid extraction from the raspberry fruit pomace. 

Considering the general lack of precautionary measures in developing countries, and that fruit and vegetable processing generates large quantities of biowaste and places a significant burden on the environment, the obtained results have both local and international significance. One can argue that the bioactive and functional molecules of raspberry fruit are already discussed in the literature and that the obtained results are based on common extraction methods. Nevertheless, due to the specific environmental conditions, this evaluation study of raspberry fruit harvested in Montenegro (in the Mediterranean region) supports the increasing body of evidence about the RFP chemical profile and biological potential. Therefore, RFP deserves to be more valorized in the future as a source of retrievable bioactive compounds and fiber, and more frequently an object of research interest in the field of nutraceuticals, food preservation, and functional food.

## Figures and Tables

**Table 1:** Total phenolic (TPC), flavonoid (TFC), and anthocyanin (TAC) content of the raspberry fruit pomace (RFP).

Type of Extraction	TPC (mg/L GAE)	TFC (mg/g QE)	TAC (mg/L C3G)
UAE	27.79 ± 1.25	8.02 ± 0.31	7.13 ± 0.43
CM	8.49 ± 1.45	3.16 ± 0.35	4.87 ± 0.38

Values represent mean ± SD of three measurements (*n* = 3). GAE—gallic acid equivalent; QE—quercetin equivalent; C3G—cyanidine-3-glucoside; UAE—ultrasound-assisted extraction; CM—conventional maceration.

**Table 2:** Data from the literature on total phenolic, flavonoid, and anthocyanin content of the RFP, whole fruit, and its products.

Species and Sample Type	TPC	TFC	TAC
*Rubus idaeus* L.			
Freeze-dried fruit [36]	2209.86 ± 70.32 mg GAE/100 g GAE	831.87 ± 12.61 mg/100 g RE	144.55 ± 0.39 mg/100 g C3G
*Rubus fruticosus*			
Lyophilized fruit [37]	N/A	6.54–24.00 mg/g RE	0.57–2.10 mg/L C3G
*Rubus idaeus* L.			
Fruit	234 ± 5.1 mg/100 g GAE	N/A	68.00 mg/100 g C3G
Pomace [19]	633.7 mg/100 g GAE	591.65 mg/100 g RE	65.21 mg/100 g C3G
*Rubus idaeus* L.			
Freeze-dried fresh fruit [38]	169 ± 4–2494 ± 77 mg/100 g GAE	N/A	10.56 ± 1.73–113.6 ± 7.7 mg/100 g C3G
*Rubus fruticosus*			
Fruit pomace [39]	7.97 ± 0.31–88.28 ±3.48 g/kg GAE	4.11 ± 0.20–45.51 ± 2.16 g/kg RE	1.14 ± 0.04–12.61 ± 0.48 g/kg C3G
*Rubus fruitcosus* Juice [40]	N/A	1.11 ± 0.04–1.20 ± 0.02 QE g/L^−1^	0.09 ± 0.01–0.12 ± 0.02 g/L
*Rubus fruticosus*			
Juice [41]	24.9 ± 1.0 mg/g GAE	N/A	5.6 ± 0.3 mg/g C3G
*Rubus idaeus* L.			
Juice [42]	164.4 ± 5.1 mg/100 g GAE	N/A	0.08 ± 0.01% C3G
*Vaccinium* spp.	171 ± 12–961 ± 15 mg/100 g GA		34 ± 1.0–515 ± 3.6 mg/100 g C3G
*Rubus* spp.	126 ± 0.3–1079 ±34 mg/100 g GAE	N/A	52 ± 0.6–627 ± 8.3 mg/100 g C3G
*Ribes* spp.	191 ± 17–1790 ± 5 mg/100 g GAE		14 ± 0.4–411 ± 12 mg/100 g C3G
Fruit [43]			
*Rubus idaeus* L.			
Frozen fruit	0.251–0.321 g/100 g GAE	N/A	0.016–0.078 g/100 g C3G
Jam [44]	0.218–0.361 g/100 g GAE		0.007–0.021 g/100 g C3G

RE: rutin equivalent; N/A: not analyzed.

**Table 3:** Antioxidant activity of raspberry fruit pomace.

Type of Extraction	FRAP (µmol/L)	DPPH		
		AAE (µmol/mL vit C Eq)	IC50 (µL/mL)	Trolox Eq (µmol/100 g Trolox Eq)
UAE	1002.72 ± 12.20	969.71 ± 8.2	20.00 ± 2.02	567.00 ± 4.56
CM	772.73 ± 10.50	931.80 ± 7.9	37.40 ± 2.43	361.27 ± 5.65

Values represent mean ± SD of three measurements (*n* = 3).

**Table 4:** DPPH and FRAP values from the literature.

Species and Sample Type	DPPH	FRAP
*Rubus idaeus* L. Pomace [51]	IC50 = 8.15–12.92 mg/mL FW	N/A
*Rubus fruticosus* L. Pomace [39]	1.03 ± 0.03–2.12 ± 0.07 mmol TEAC g^−1^	N/A
*Rubus idaeus* L. Fruit extract [52]	EC50 = 31.5 mg/cm^3^	10.08 mmol Fe^2+^/kg RM
Various fresh fruits [53]	N/A	1460–15,940 μmol/kg FW

**Table 5:** Content of polyphenols in raspberry fruit pomace.

µg/g Extract												
**Type of** **Extraction**	**Galic Acid**				**Caffeic Acid**				***p*-Coumaric Acid**			
	**Met 1**		**Met 2**		**Met 1**		**Met 2**		**Met 1**		**Met 2**	
	**x**	**u**	**x**	**u**	**x**	**u**	**x**	**u**	**x**	**u**	**x**	**u**
UAE	8.75	1.31	7.92	1.19	19.17	0.96	17.36	0.87	0.56	0.06	0.51	0.05
CM	6.36	0.95	5.69	0.85	9.95	0.50	8.90	0.45	0.95	0.09	0.85	0.08
**Type of Extraction**	**Quercetin**				**Chlorogenic Acid**				**Ellagic Acid**			
	**Met 1**		**Met 2**		**Met 1**		**Met 2**		**Met 1**		**Met 2**	
	**x**	**u**	**x**	**u**	**x**	**u**	**x**	**u**	**x**	**u**	**x**	**u**
UAE	0.06	0.00	0.06	0.00	3.56	0.18	3.22	0.16	105.52	4.22	95.59	3.82
CM	1.27	0.02	0.24	0.02	1.55	0.08	1.38	0.07	55.00	2.2	49.15	1.97

Met 1: non-hydrolyzed sample; Met 2: hydrolyzed sample; x—quantified value; u—expanded measurement uncertainty calculated using coverage factor k = 2.
